# The feasibility of health professional student delivered social visits for stroke survivors with loneliness

**DOI:** 10.3389/fstro.2024.1393197

**Published:** 2024-05-15

**Authors:** Jason Burnett, Jordan Broussard, Bronson Ciavarra, Louisa Smitherman, Mary Li, Emma Thames, Sharon Zachariah, Grace Kim, Rachel Pijnnaken, Hannah Zeller, John Halphen, Sean I. Savitz, Namkee Choi, Jennifer E. S. Beauchamp

**Affiliations:** ^1^McGovern Medical School, University of Texas Health Science Center at Houston, Houston, TX, United States; ^2^Institute for Stroke and Cerebrovascular Diseases, University of Texas Health Science Center at Houston, Houston, TX, United States; ^3^Center for Nursing Research, Cizik School of Nursing, University of Texas Health Science Center at Houston, Houston, TX, United States; ^4^Cizik School of Nursing, University of Texas Health Science Center at Houston, Houston, TX, United States; ^5^Department of Neuroscience, The University of Texas at Austin, Austin, TX, United States; ^6^Department of Neurology, McGovern Medical School, University of Texas Health Science Center at Houston, Houston, TX, United States; ^7^Steve Hicks School of Social Work, The University of Texas at Austin, Austin, TX, United States

**Keywords:** stroke, loneliness, social isolation (SI), student-led, social connectedness

## Abstract

**Objectives:**

To examine the feasibility of a social phone call program to address social isolation and loneliness in stroke survivors.

**Materials and methods:**

We paired 14 lonely community-living stroke survivors with 14 health professional students for 6-weekly unstructured social phone calls. Feasibility data and measures of social isolation, loneliness and other psychosocial metrics were collected pre- and post-intervention. Students journaled following each unstructured call to capture the informal conversation and their sentiments.

**Results:**

Sixty-two percent of the targeted sample was interested. Fourteen eligible and interested participants were enrolled. The 13 (93%) participants completing all calls and surveys were an average of 57 years old, 85% female, and 77% non-Hispanic white. At baseline, participants were highly lonely and moderately depressed. Participants disclosed physical and emotional challenges, previous valued employment, and enjoyment from the calls. Students reported enjoying the connections, learning about the struggles of aging-in-place after stroke, and valuing compassionate care for the stroke population.

**Conclusions:**

Knowledge gaps remain regarding effective social support interventions to provide continuity of care directed at managing social disconnection after stroke. A health professional student-delivered social phone call intervention with stroke survivors appears to be a feasible, in part, and encouraging approach for addressing social isolation and loneliness. Future trials require re-evaluation of eligibility criteria and strategies to boost enrollment before efficacy testing is conducted in a larger trial.

## Introduction

Cerebrovascular accident or stroke is a serious medical condition that can increase risks for cognitive impairment and functional disabilities, depression and anxiety, social disconnection, and mortality (Towfighi et al., 2017; Tsao et al., 2023). Approximately 800,000 people in the U.S., a disproportionate share of them being low-income or racial/ethnic minorities, experience stroke each year (Tsao et al., 2023). Social isolation and loneliness (SIL) are important modifiable risk factors that increase the risk of stroke (32%) and recurrent stroke (40%) and contribute to poor outcomes including depression and anxiety (Valtorta et al., 2016; National Academies of Sciences, 2020; Cené et al., 2022). Interventions such as friendly visits, intergenerational and peer-initiated social phone calls, and behavioral activation programs have been effective in reducing SIL in older adults (Choi et al., 2020; Bruce et al., 2021; Hoyumpa et al., 2022; Noble et al., 2022; Fields et al., 2023). However, these interventions have not been tested in stroke survivors (Cené et al., 2022). Given the positive cognitive, social, and health effects of intergenerational engagement on older adults, we examined the feasibility of a health professional student (HPS)-delivered social phone call intervention with community-living, lonely adults who had experienced stroke. HPS open-ended journal entries following each unstructured call were also explored.

## Materials and methods

### Study design, target population, and sample recruitment

We conducted a 6-week, single group, pretest-posttest study. The target population was community-dwelling adult stroke survivors 18 years and older receiving care from an outpatient academic-affiliated stroke clinic in a large metropolitan area. Using convenience sampling, a trained research coordinator approached potential participants during their scheduled clinic visits. A two-step screening and recruitment process was utilized. First, the research coordinator reviewed the stroke clinic's scheduled visit log on recruitment days and conducted a pre-screen of the electronic health records (EHR) to identify patients who met the age and history of ischemic and hemorrhagic stroke eligibility criteria. Second, those meeting the criteria were approached about the study and if agreeable assessed for the remaining eligibility criteria: (1) scored >4 on the 3-item University of California, Los Angeles (UCLA) loneliness scale (Hughes et al., 2004), (2) could read, write, and speak English, (3) provided written informed consent, and (4) agreed to be available for 6 consecutive weekly telephone calls from a HPS student lasting up to 1 h in duration. Exclusion criteria were: (1) living outside of the home (e.g., in-patient rehabilitation center); (2) had EHR documentation of moderate to severe cognitive impairments; and/or (3) had aphasia.

### Health professional student recruitment

HPS volunteers were recruited from the study-affiliated medical and nursing schools and an undergraduate pre-medical program. Medical students were recruited through an online medical student social group where the study flier was distributed by a medical student not affiliated with the study. Nursing students were solicited through internal emails sent by a study principal investigator affiliated with the school of nursing. An undergraduate pre-medical student was recruited given his or her expression of interest in a summer volunteer opportunity.

### Dyad matching and calling schedule

Each participant was randomly assigned to a HPS who then initiated 6-weekly social phone calls. Prior to initiating any calls, students were required to complete online Good Clinical Practice and Human Subjects Research trainings and received 30-min pre-recorded training on empathy-based communication (Peisachovich et al., 2021; Moudatsou et al., 2020) as well as 30-min live training by a psychologist on identifying and responding to suicidal ideation. A suicide safety protocol was included in each Research Electronic Data Capture (REDCap) data entry form accessed by students for each call in case of mental health emergencies (Harris et al., 2009, 2019).

All calls were initiated within 1 week of baseline data collection. Each call was scheduled for 1 h with flexibility to be shorter or longer depending on the dyad (HPS and participant) availability and preferences. Calls were purposely unstructured in order to allow the HPS and participant flexibility to discuss what they wanted (e.g., daily activities, health care appointments, family, and events) and to allow the development of meaningful connections. If the HPS was unable to reach the participant after three call attempts, the research coordinator attempted two additional calls before the participant was considered lost to follow-up.

### Data collection

Immediately following consent, the research coordinator sent an email to each enrolled participant containing the REDCap link with the baseline demographic, and biopsychosocial assessments (Harris et al., 2009, 2019). Stroke type, date, and severity, and comorbid conditions were obtained from the EHR. The Charlson Comorbidity Index was used to catalog comorbidities. The standardized biopsychosocial measures, also assessed at 6-weeks using an emailed REDCap link, were the: (1) 20-item UCLA Loneliness Scale (Russell, 1996), (2) 10-item Duke Social Support Index (10-item DSSI) (Wardian et al., 2013), (3) 20-item Center for Epidemiologic Studies Depression Scale (CES-D) (Radloff, 1977) and the (4) 7-item Generalized Anxiety Disorder 7 (GAD-7) (Spitzer et al., 2006). Social isolation and loneliness were the primary outcomes. For each call, HPS documented in REDCap: (1) call length, (2) days and times of each call, and (3) open-ended journal entries describing the call conversations and any additional personal sentiments (Harris et al., 2009, 2019). Open-ended journal entries were used to capture call content and HPS sentiments. To help illustrate the HPS experience across disciplines, we provided two narrative case examples (1 medical HPS and 1 nursing HPS) summarizing their six calls and what they learned from the experience. The University of Texas Health Science Center at Houston (UTHealth), Committee for the Protection of Human Subjects approved the study.

### Analysis

Descriptive statistics were used to characterize the data using frequencies, means, standard deviations, counts, and proportions. The Reach, Effectiveness, Adoption, Implementation and Maintenance (RE-AIM) framework guided the analysis using available data (Glasgow and Estabrooks, 2018). Reach was assessed by dividing the number of eligible participants that participated in the study by the total number of eligible participants. For this study, eligibility was defined as those who met the criteria of the full eligibility screening. Although not powered to assess effectiveness, pre-test and post-test scores were computed for all study assessments to assess trends. Implementation was evaluated based on the proportion of (1) completed study phone calls, (2) participants completing all baseline and follow-up assessments, and (3) HPS completed journal entries. Attrition was determined by dividing the number of participants who completed the study by the number of participants enrolled. Enrollment was defined by consenting and completing the baseline assessments. Study completion was defined as completing baseline and post-intervention surveys and all 6 HPS-participant dyad calls. Feasibility was characterized based on participant enrollment rate, number of calls completed, and attrition rate. To identify common HPS call experiences and sentiments, the HPS journal entries were explored to describe the social visits.

## Results

### Prescreen demographics

**Figure 1:**
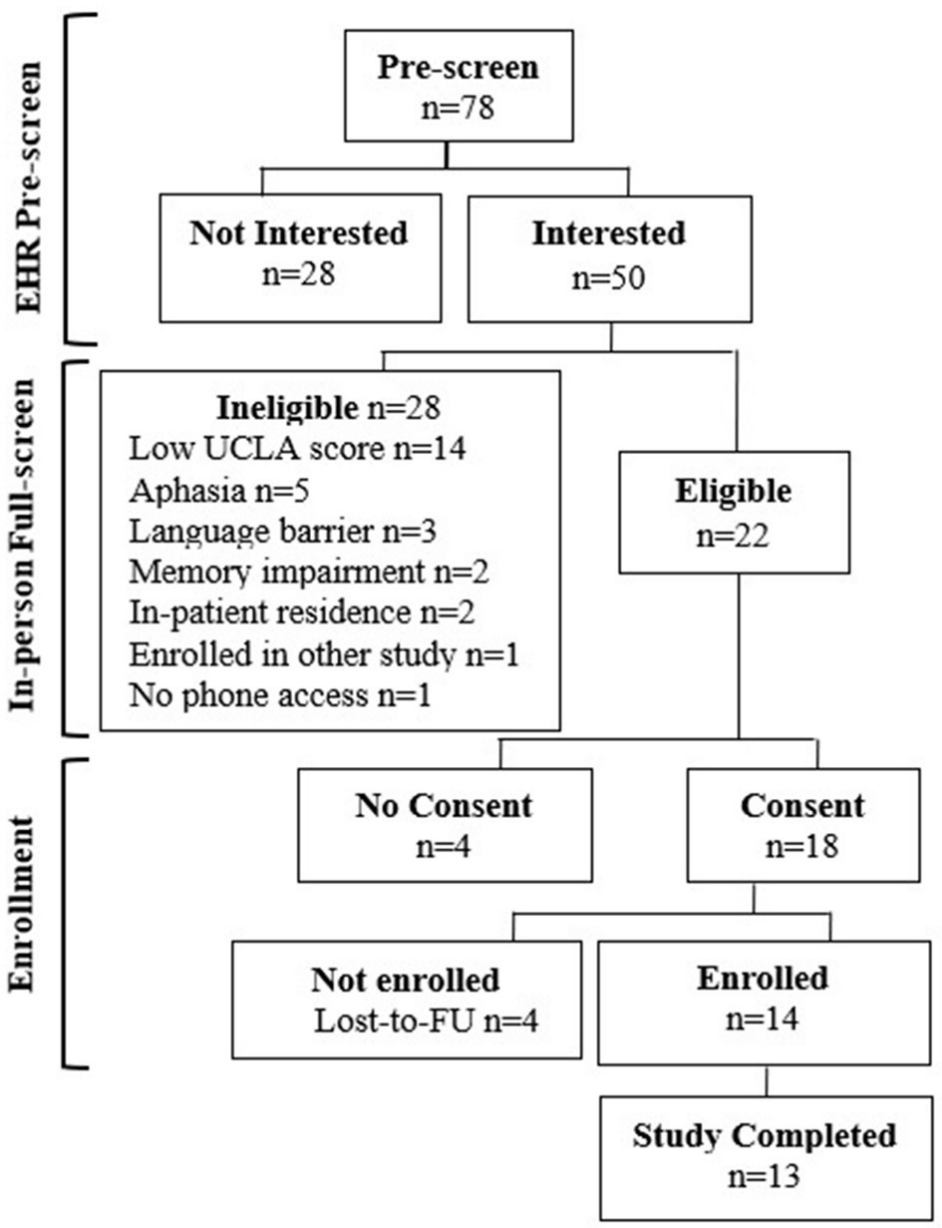
Study flow diagram.

Figure 1 diagram the study prescreen, full screen, and enrollment and retention numbers. Seventy-eight adults were determined to be eligible for the full screen based on the EHR prescreen. The average age of this group was 61 + 16 years. The majority of prescreened individuals were female (54%), and 33% were Black, 46% were White, and 85% were non-Hispanic adults. Fifty (78%) of the 78 adults approached were interested and completed the full screen. There was a 1-year difference in the mean age between those agreeing to the full screen and those not interested. Males (66%) were more likely than females (61%) to agree to the full screen and White individuals (69%) were more likely than Black individuals (58%) to agree.

### Full screen demographics

Based on the full screen, 22 adults (44%) were eligible for enrollment and 28 (56%) were ineligible due to low UCLA short scores, aphasia diagnoses, language barriers, issues with memory, or living situations. Eighteen (81%) eligible participants consented to the study. The average age of consenting individuals was 58 ± 14 years. The majority of consenting adults were female (78%), and 61% were White, 39% were black, and 94% were non-Hispanic adults. Four individuals were lost to follow-up after providing consent and did not enroll in the study, and four other individuals were lost to follow-up before providing consent resulting in a pre-enrollment attrition rate of 36%.

### Enrollment and reach

Overall, 14/22 (64%) of the eligible participants were enrolled in the study and 13 (93%) completed the study. Table 1 provides the demographic characteristics of the 13 adults completing the study. Briefly, the mean participant age was 57 years (±14.3), 77% of participants were White non-Hispanic, and 85% were female. The median time since stroke was 12 months.

**Table 1:** Sample characteristics of *N* = 13 adults who experienced stroke and reported loneliness and completed the study.

**Characteristics**	**Participants^a^**
Age in years, mean (SD)	57 (±14.3)
Female gender	11 (84.6)
**Race/ethnicity**	
White non-Hispanic	10 (76.9)
Black non-Hispanic	3 (23.1)
**Highest level of education**	
High school graduate, GED, or less	4 (30.8)
Some college, no degree	1 (7.7)
College degree	8 (61.5)
**Current employment status**	
Employed	2 (15.4)
Unemployed	10 (76.9)
Other	1 (7.7)
**Marital status**	
Married or domestic partnership	8 (61.5)
Single	5 (38.5)
**Number of people in household**	
2	8 (61.5)
3	3 (23.1)
4	2 (15.4)
**Stroke type**	
Ischemic	10 (77)
Hemorrhagic	3 (23)
**Post-stroke time frame, median**	12 months
**Stroke severity**	
NIHSS,^b, e^ median (SD)	4 (±8.3)
**Stroke disability**	
mRS,^c, e^ median (SD)	2 (±1.7)
**Comorbid conditions** ^e^	
CCI,^d^ median (SD)	3 (±1.7)
Myocardial infarction	1 (7.7)
Congestive heart failure	1 (7.7)
Cerebrovascular disease	1 (7.7)
Uncomplicated diabetes mellitus	3 (23.1)
Hemiplegia	2 (15.4)
Solid tumor (non-metastatic)	1 (7.7)

^a^Values in n (%) unless otherwise indicated; ^b^NIHSS, National Institutes of Health Stroke Scale; ^c^mRS, modified Rankin scale; ^d^CCI, Charlson Comorbidity Index; ^e^Values may not include data for all 13 participants due to restricted Electronic Health Record access.

Fourteen HPS volunteered for the study. Most HPS were female (77%) and enrolled in medical school (77%). Medical students were equally distributed across health professional training years 1-3 and nursing students were equally distributed across training years 3 and 4.

### Effectiveness

Overall, there were minimal improvements on the targeted outcomes with the largest improvement (e.g., a decrease of 4-points from baseline to post-intervention) occurring for loneliness. However, all of the outcome score changes trended toward improvement. Table 2 provides pre-test and post-test assessment data.

**Table 2:** Psychosocial measurement scores of *N* = 13 adults who experienced stroke and reported loneliness and completed the study.

**Psychosocial measures**	**Pre-intervention scores**	**Post-intervention scores**
UCLA loneliness scale^a^	47.77 (11.9)	43.92 (13.78)
Center for epidemiologic studies of depression^b^	19.77 (12.1)	18.77 (10.9)
Generalized anxiety disorder-7^c^	7.54 (4.7)	6.62 (4.7)
Duke social support index^d^	22.15 (3.8)	21.92 (3.9)

Scores presented as mean values and standard deviations. Score interpretations. ^a^Scores range from 20-80, < 28 no or low loneliness, 28-43 moderate loneliness,>43 high. ^b^Scores range from 0 to 60, ≥16 indicates risk for clinical depression. ^c^Scores range from 0-21, 0-4 minimal anxiety, 5-9 mild anxiety, 10-14 moderate anxiety, ≥15 severe anxiety. ^d^Scores range from 10-30, higher scores indicate more social interaction and support.

### Implementation

Seventy-nine (94%) of the expected calls and 78 (92%) of the expected HPS journal entries were completed. The only calls and missing journal entries came from the one participant and student dyad that dropped from the study. The average phone call lasted 49 ± 14 min with a range of 10-96 min.

### HPS experiences

Student journaling revealed that most of the participants disclosed physical and emotional challenges related to their stroke experience. The majority of stroke survivors discussed their previous or current employment/careers, and all HPS students noted, in at least one of their journal entries, that the participant seemed to be enjoying the calls. The weekly journaling showed that 100% of the HPS enjoyed the weekly calls and the major benefits of the phone calls were learning about post-stroke challenges such as mobility, SIL, depression, and valuing holistic compassionate stroke care. See Supplementary Table 1 for a sample of HPS journal quotes.

### Narrative case example: nursing student experience

The stroke survivor expressed a variety of psychosocial concerns. These included social disconnection, perceived physical, emotional, financial and time constraining burdens for her daughter, a personal loss of community value, and helplessness. Most were related to the survivors' activities of daily living impairments and full dependence on her daughter for safety and care. With her daughter being a full-time worker and part time college student, the survivor felt she was a continual cause of emotional stress and financial burden. The survivor reminisced about the personal value of her prior work helping homeless young adults. She stated that she often feels alone and incapable of helping others when she is around those she loves. Nevertheless, the survivors mood seemed to improve during our calls.

My experience left me with the feeling that I was truly impacting the mental health of a stroke survivor and it left a profound mark on how I will approach patient care as a future nurse. Central to this approach is recognizing the psychological, emotional, and social impacts of stroke to deliver a holistic approach to patient care including promoting meaningful connections. Recognizing the impact of stroke on relationship dynamics and the importance of positive social interactions for recovery, I will also extend support to the family members and caregivers who play pivotal roles in the care process and encourage my patients to participate in community support groups and connect with peers who have faced similar challenges as well as others.

### Narrative case example: medical student experience

I experienced the significant healing power and freedom that comes from open, genuine and active communication between two strangers willing to share vulnerabilities. The stroke survivor shared personal values and beliefs and challenges of stroke survivorship including feeling lonely, being socially isolated, and disconnected from community. He shared how his stroke has largely forced him to stay indoors and rarely leave the house, now unable to greet strangers and make them happy which provided him with a sense of meaning and purpose. As our conversations progressed, the participant shared that speaking about his stroke at home is taboo and the opportunity to speak with someone in an open and honest way was invaluable and comforting. Despite these struggles, he has used his stroke experience to provide wisdom and support for others who have experienced strokes.

The participant continuously encouraged me to think of him as my first patient. He left me feeling encouraged and supported. In light of this relationship structure, the participant made explicit efforts to share the keys to being the best possible physician to my patients—the most important being, to first be the best possible person I could be. My experience taught me it is critical to make fervent efforts to know the patient as a person and not merely by their medical pathology. This atmosphere was present during every conversation with the participant, and I realized that healing did not only occur for the participant, but it occurred for me as well.

## Discussion

The current study piloted a SIL intervention in adult stroke survivors using weekly HPS-delivered social phone calls. Friendly-caller programs are supported methods to address SIL (Kahlon et al., 2021; Hoyumpa et al., 2022), but the feasibility and effectiveness of these programs are lacking for the stroke population. Our study found that most stroke survivors were interested in participating, but black individuals were somewhat less likely to agree to participation. We also discovered a high ineligibility rate among targeted stroke survivors therefore warranting examination. Encouragingly, enrollment of eligible stroke survivors and program implementation processes were successful and trends toward intended intervention effects were identified. Implications of the current study findings and considerations for future studies are presented below.

The findings support the feasibility of reaching and engaging the adult stroke population using a HPS-delivered social phone calls intervention, but strategies are needed to support recruitment and participation of black stroke survivors. Sixty-two percent of the targeted population expressed interest in the program, however, black stroke survivors were less likely to participate. We did not capture reasons for refusal, but literature on lower research participation by black individuals cites higher distrust in research, especially in absence of prior clinical research experience (Braunstein et al., 2008). This holds true even among those who are actively engaged in the healthcare system. Distrust in the healthcare system may be high (Durant et al., 2011), but medical care may be viewed as a necessity, especially for stroke recovery, whereas participating in research may be viewed as an unnecessary risk. Alternatively, black stroke survivors are reported to have higher socioeconomic barriers, higher stroke-related physical and cognitive deficits, higher incidents of post-stroke depression, and poorer post-stroke recovery compared to non-Hispanic White stroke survivors (Magwood et al., 2019). These can all impact research participation decisions. Future SIL studies should explore the reasons for participation and non-participation among black stroke survivors to support the development of appropriate recruitment strategies.

The ineligibility rate was high and may need to be re-evaluated to increase the opportunity for interested stroke survivors to benefit from social engagement interventions. Screening for loneliness may be met with resistance or concealment due to stigma and embarrassment. Moreover, short loneliness screens such as the 3-item UCLA scale may not be sensitive enough in the stroke population. In our study, 50% of interested participants were ineligible due to not meeting the loneliness criterion. This was especially true for males and accounts for lower male participation despite 46% of males and 52% of females stating interest in participating. Perhaps expressing interest in receiving a friendly social phone call is indicative of being lonely and potentially benefiting from a SIL intervention. Future studies should explore whether expressed interest correlates with long-version loneliness scales and intervention benefit. The other criteria such as aphasia, living situation, memory, and language barriers could be overcome in a larger funded study where videoconferencing could be used, caregivers could be recruited to help coordinate calls, and bi-lingual HPS could be recruited.

Although underpowered to assess statistical effectiveness of the social calls, the assessment data indicated encouraging trends with the largest effect found for loneliness. Social phone calls are designed specifically to alleviate loneliness, but have been shown to also improve outcomes such as depression and anxiety given their relationship with social disconnection (National Academies of Sciences, 2020; Kahlon et al., 2021; Hoyumpa et al., 2022). This is important because there are few studies targeting social connection in stroke survivors despite a 40% increased risk in recurrent stroke predicted by social disconnection (e.g., SIL) (Cené et al., 2022).

Moreover, a SIL intervention that decreases post-stroke depression and anxiety could provide an alternative or adjunct approach to commonly used and sometimes minimally effective or harmful pharmaceutical therapies (Lee et al., 2021). Larger more well-powered studies are needed to understand the effects of HPS-led social phone call interventions and other SIL interventions on post-stroke biopsychosocial outcomes that impact recovery, morbidity, and mortality.

The intervention implementation was high and should encourage future experimental studies of SIL in this population. Often, stroke survivors suffer cognitive, emotional, and physical changes which may cause reluctance or challenges to socially engage or participate in research studies (Towfighi et al., 2017; Byrne et al., 2021). Perhaps the remote nature of the social phone calls made it easier and more acceptable for stroke survivors to socially engage due to changes in appearance, social abilities, and social identify. One stroke survivor stated that after her stroke, her pain was debilitating, noticeable, and disruptive, causing her to self-isolate and avoid others at home and at work. Regarding the ability to provide data, 93% of stroke survivors individually completed all of the pre and post assessments indicating the ability to fill out the survey data electronically. Other social visit modalities warrant further exploration in this population to determine the types of acceptable approaches for meeting stroke survivor's social needs and desires. Perhaps the flexibility in the timing of the calls, the remote nature, and length of time planned for each call fit well with HPS learning schedules making recruitment and retention practical (Ali et al., 2021).

It is important to briefly highlight the experience of HPS. Exploratory review of the HPS journals revealed that all HPS enjoyed the calls and the study raised stroke awareness including the importance of the social context and offered learning opportunities for supporting persons who experienced stroke as future health care professionals. A student reflected, “I think that this should be something done nationwide as a way to help students learn the art of listening and compassionate communication while also getting the opportunity to take part in something bigger than themselves.” Other volunteer programs have reported similar deep learning experiences for HPS, but none have targeted stroke survivors (Haidar et al., 2020; Ali et al., 2021; Siqueira et al., 2022).

Several limitations of this study should be noted. First, given low study funding, a full program evaluation using the RE-AIM framework was not possible. The pilot nature of this study did not allow for evaluating the adoption and maintenance components or statistically evaluating effectiveness. Future SIL studies in this population should be designed to adequately address the RE-AIM framework components. Relatedly, a small convenience sample with no control group was used which limits the evaluation of effectiveness and generalizability of the findings. Nevertheless, the recruitment and completion rates as well as the qualitative data suggest that a larger study could be feasible. Formal qualitative analyses may benefit future studies especially among stroke participants. No formal assessments of acceptability beyond retention were assessed which could inform future study designs. The exclusion of Spanish only speaking adults is also a limitation that can be overcome in future studies to understand the feasibility and acceptability of this approach in health disparity populations. Moreover, sampling for more stroke clinics treating a wider age range of stroke survivors may help recruit a more age-diverse sample that could benefit from the intervention.

This study has implications for future HPS-delivered psychosocial interventions with stroke survivors. While, friendly visits (e.g., social phone calls) are effective at reducing loneliness and depression in vulnerable populations, manualized approaches such as behavioral activation (BA) interventions, are likely to create more positive outcomes especially for interventions as brief as six sessions (Choi et al., 2020; Pepin et al., 2021). In fact, a brief layperson-delivered BA, adapted to target social connection in homebound older adults with similar impairments as stroke survivors, significantly reduced SIL and depression (Bruce et al., 2021), offering a potential mechanism to address post-stroke depression, a common and debilitating complication of stroke. If optimized to fit the flexibility of their learning schedules, HPS could be an untapped resource for delivering manualized BA approaches to address the SIL and post-stroke depression nationally.

This study provides preliminary evidence supporting the ability to intervene in the stroke population to address SIL using remote social visits from HPS. Future studies should utilize the RE-AIM framework during the planning phase to improve data collection and program evaluability. More studies are needed to determine ways to improve recruitment of stroke survivors, especially black individuals, Spanish-speaking populations, and men to power studies assessing changes in psychosocial effects of these HPS and stroke survivor social engagement. Nevertheless, the model provided in this study offers potential low-cost and scalable way to address SIL and related mental health factors such as post-stroke depression and anxiety in persons who have experienced stroke. Moreover, it may be a model for raising HPS stroke awareness and building their compassion for the plight of persons who have experienced stroke. More well-designed and generalizable studies are necessary, especially those using more social contacts and/or brief manualized evidence-based approaches such as behavioral activation.

## Data availability statement

The raw data supporting the conclusions of this article will be made available by the authors, without undue reservation.

## Ethics statement

The studies involving humans were approved by the UTHealth Houston Committee for the Protection of Human Subjects Institutional Review Board. The studies were conducted in accordance with the local legislation and institutional requirements. The participants provided their written informed consent to participate in this study.

## Author contributions

JBu: Conceptualization, Methodology, Visualization, Writing – original draft, Writing – review & editing, Formal analysis, Investigation, Resources, Supervision, Validation. JBr: Writing – original draft, Writing – review & editing. BC: Data curation, Writing – original draft, Writing – review & editing, Investigation. LS: Data curation, Writing – original draft, Writing – review & editing, Investigation. ML: Data curation, Writing – original draft, Writing – review & editing, Investigation. ET: Data curation, Writing – original draft, Writing – review & editing, Investigation. SZ: Data curation, Writing – original draft, Writing – review & editing, Investigation. GK: Data curation, Writing – original draft, Writing – review & editing, Investigation. RP: Data curation, Visualization, Writing – original draft, Writing – review & editing. HZ: Data curation, Writing – original draft, Writing – review & editing, Investigation. JH: Data curation, Writing – original draft, Writing – review & editing, Investigation. SS: Writing – original draft, Writing – review & editing, Conceptualization, Resources. NC: Writing – original draft, Writing – review & editing. JB: Conceptualization, Formal analysis, Funding acquisition, Investigation, Methodology, Project administration, Supervision, Visualization, Writing – original draft, Writing – review & editing, Resources, Validation.
