# Supplementary material for: The feasibility of health professional student delivered social visits for stroke survivors with loneliness
Source: Front Stroke. 2024 May 15;3:1393197. doi: 10.3389/fstro.2024.1393197 (PMC12802625; doi:10.3389/fstro.2024.1393197)
Supplement: Supplementary file 1 [file Table_1.DOCX]

Supplement 1: Health Professional Student Reflection Quotes

| I think that this should be something done nationwide as a way to help students learn the art of listening and compassionate communication while also getting the opportunity to take part in something bigger than themselves. |
| --- |
| We covered mundane topics and dove into deep cuts as well. I only wish it was in person to facilitate that secondary level of communication. Given that stroke patients often have difficult times with speech, he often became frustrated with his speech and I think it affected his mood. |
| Through these conversations, I've gained a profound perspective and understanding of the challenges and triumphs faced by stroke patients. Hearing their stories firsthand, their struggles to regain mobility, communication skills, and overall independence, has allowed me to see beyond the surface and truly empathize with their journey. |
| These interactions have illuminated the strength of the human spirit, the power of resilience, and the importance of social support in the face of adversity. Engaging in these conversations has not only broadened my knowledge about the medical and emotional aspects of stroke recovery but has also deepened my appreciation for the small, everyday victories that we often take for granted. This study has shown me the significance of empathy and connection in fostering healing and personal growth, ultimately shaping me into a more compassionate and understanding individual. |
| I definitely perceived our conversations as more therapeutic for the participant—it became obvious that their diagnosis and experience with stroke weighed heavily on their mind and I was a safe listener they could talk to. |
| I think some parts of me would’ve liked the video-call option, as for some people it can make the study more personable. |
| Often in the pediatric population, I rarely get to interact with elderly patients. I was able to see similarities in mental health between both populations struggling with a life changing medical diagnoses. |
| Coming into my first year of medical school, I had trouble finding a balance between studying and just living my life. I spent many days and nights studying alone at home, feeling confined by space and time. When this study came around, I felt it was a great way to pursue my interests in psychiatry, but what I got out of it was more personal. |
| Getting to talk to my assigned partner was a treat each week I had to call her! She was chatty and very pleasant to talk to. I found it became a bright spot in my days of isolation. |
| Getting to talk to her helped me see where the similarities and differences lie between our age groups and how I can interact and relate to those much older than me. I appreciate that this study had hidden little lessons for me that I hadn’t expected when walking into it. I know feel I have a better understanding of what stroke survivors experience in day to day lives. And I feel I can better communicate with elderly populations. This has prepared me well for opening myself up to others in the coming years of my career, be it with colleagues or my future patients! |
| I believe conducting the study over the phone fostered participant openness. |
| This study highlighted the significance of being present and purposeful with our time set aside for the phone call. For example, setting aside the laptop and laundry, and putting all the focus and attention on the conversation with the participant. I found this to be very significant and it is something I will bring into the clinical setting. I believe this study holds potential for enhancing clinical practices, particularly in preparing patients for life post-hospitalization. This study shed light on the long-term impacts of stroke and how it affects various aspects of life such as social interactions, family roles, intimate relationships, and caregiving dynamics. I also found this study to show the need for more resources that support people post-stroke. |
| I oftentimes found myself struggling to come up with what to say next after we finished a topic, but thankfully she was very talkative and would jump to another topic. I found my role as more of an empathetic listener who was there to hear her story, but she was also interested in mine. |
| Social Phone Calls with Stroke Patients is an enriching study that allows you to connect with a complete stranger and become a part of their life in a short amount of time. The depth of connection you build in a truncated time is both rewarding and challenging. |
| It was difficult to hear that they were going through such difficult times, and I could do nothing but listen from afar... It was an eye-opening experience of the backgrounds and types of patients you might encounter in healthcare, but also in life. |
